# Molecular Detection Methods in HPV-Related Cancers

**DOI:** 10.3389/fonc.2022.864820

**Published:** 2022-04-27

**Authors:** Jordana Williams, Morris Kostiuk, Vincent L. Biron

**Affiliations:** Division of Otolaryngology-Head and Neck Surgery Research Laboratory of Alberta, Department of Surgery, University of Alberta, Edmonton, AB, Canada

**Keywords:** human papillomavirus, diagnostic tools, p16, droplet digital polymerase chain reaction, polymerase chain reaction, immunohistochemistry, *in situ* hybridization

## Abstract

Human papillomavirus (HPV) is responsible for most cervical cancers and some head and neck cancers, including oropharyngeal squamous cell carcinoma and sinonasal carcinoma. Cervical cancer is commonly diagnosed by liquid-based cytology, followed by HPV testing using commercially available DNA polymerase chain reaction (PCR), p16 immunohistochemistry (IHC), or DNA/RNA *in situ* hybridization. HPV in head and neck cancers is commonly diagnosed by p16 IHC or by RT-qPCR of HPV-16 E6 and E7 oncoproteins. Droplet digital PCR has been reported as an ultrasensitive and highly precise method of nucleic acid quantification for biomarker analysis and has been used to detect oncogenic HPV in oropharyngeal and cervical cancers.

## Introduction

Human papillomavirus (HPV) is the most common sexually transmitted infection (STI) in the world (1). and classified as a carcinogenic infectious agent by the International Agency for Research on Cancer (2). Both sexually active men and women will be infected at least once without developing any symptoms or cancerous diseases in their lifetime (1). However, only some HPV strains are oncogenic. These have been shown to cause most cervical cancers and some head and neck cancers, particularly in the oropharynx (3, 4) and, to a lesser extent, in the sinonasal region (5). HPV testing is important clinically for the accuracy of diagnosis, patient-centered treatment, and prognostication (3, 6–11).

Cervical cancer screening and diagnosis is minimally invasive. It combines liquid-based cytology stained Papanicolaou stain (Pap smear) and HPV testing using DNA/RNA PCR-based methods (12, 13). The association between cervical squamous cell carcinoma (CSCC) and HPV is well established, as HPV is known to cause most cervical cancers (1–3, 12, 13). In developed countries, cervical cancer has been effectively controlled by cytological screening, which involves physician-administered cervical samples and directed cervical exams which are interpreted by a trained cytopathologist. However, in low- and middle-income countries where the burden of cervical cancer is the highest (1, 2), such established screening programs are not available nor feasible. Some of the barriers that affect the success of the screening programs include the availability of physicians, trained personnel that can interpret the sample results, access to equipment and technology, and social and cultural issues (14). To overcome these drawbacks, recent studies have investigated the use of self-sampling swabs for HPV detection to replace Pap smears and cervical exams as first-line screening. Their results showed that self-sampling has greater sensitivity compared to traditional cytology and similar sensitivity to clinician-collected specimens (14–16). The studies suggested that self-sampled HPV testing can be cost-effective and can be used as a primary screening strategy or in addition to existing screening programs. By self-sampling, the cost of testing can be lowered and the level of screening attendance will be increased, and it can attract long-term under-screened women or never-screened women to participate (17). However, the HPV assays that have been developed have limited sensitivity, specificity, and replicability in resource-limited settings (12, 13, 18).

For head and neck cancers, p16INK4a (p16) immunohistochemistry (IHC) is a widely used surrogate marker for oncogenic HPV (19, 20). Since HPV-related SCC in the head and neck region is predominantly seen in the oropharyngeal zone, p16 IHC testing is considered an acceptable clinical standard for the diagnosis of oropharyngeal SCC. Although sinonasal SCC is thought to be associated with HPV in many cases, p16 or direct HPV testing is not routinely done for these cancers (21, 22). Most methods of HPV detection in head and neck SCC, including p16 IHC, require a fine needle aspirate (FNA) or tissue biopsy (19, 20). This can often be limiting because special equipment is needed to acquire FNA samples and tissue biopsies are often invasive and resource-intensive, because special equipment is needed to acquire FNAsamples and they are obtained under general anesthesia.

Droplet digital polymerase chain reaction (ddPCR) is a promising technology for the minimally invasive detection of oncogenic HPV. It allows for the quantification of the absolute amount of target nucleic acid present with high precision and reproducibility (23). ddPCR involves partitioning a single nucleic acid sample into up to 20,000 uniform, nanoliter-sized water-in-oil droplets, amplifying them by PCR, analyzing each droplet individually, and reporting the results digitally (23, 24). This method quantifies the absolute amount of target nucleic acid present with high accuracy and reproducibility that is several orders of magnitude higher than traditional PCR (23, 24). ddPCR is a highly sensitive method for the identification of oncogenic HPV as it is able to quantify gene expression with extremely low copy numbers (25–27). This method can be applied in the early detection of oncogenic HPV in swabs from the oropharynx, sinonasal, and cervix.

## Epidemiology

HPV infection is recognized as one of the major causes of viral-related cancers in both men and women. It is classified into two categories: low-risk HPVs (LR-HPVs), which are responsible for skin warts on the hands, feet, and around the genitals and the anus, and high-risk HPVs (HR-HPVs) associated with anogenital (cervical, anal, vulvar, vaginal, and penile) and head and neck cancers (mainly oropharyngeal and sinonasal) (1). There are more than 200 genotypes of HPV, but only a few are considered carcinogenic. There are as many as 15 HR-HPV types (HPV types 16, 18, 31, 33, 35, 39, 45, 51, 52, 56, 58, 59, 68, 73, and 82), and globally, HPV 16 is the most frequent oncogenic type (1–4). It is estimated that 4.5% of all cancers worldwide (630,000 new cancer cases per year) are attributable to HPV infection: 8.6% in women and 0.8% in men. Presented in **Table 1** is a summary of the epidemiology of HPV-associated Cervical cancer, OPSCC and Sinonasal carcinoma.

**Table 1:** Epidemiology summary of HPV-associated cervical cancer, OPSCC and sinonasal carcinoma.

	Cervical cancer	OPSCC	Sinonasal carcinoma
Incidence	Decreasing	Increasing	Decreasing
Prevalence	Higher in developing countries	Higher in developed countries	Higher in developed countries
Sex	100% female	>70% male	Male and female about similar rates
Age	Under 50	Under 60	50s
Etiology	Almost all are caused by HPV	Tobacco and alcohol remain important causes, along with HPV	Environmental toxins such as tobacco and wood dust, *etc.*, along with HPV
HPV genotype	50% HPV16, 20% HPV18	>90% HPV16, HPV18	82% HPV 16, 12% HPV 31/33, 6% HPV 18

Cervical cancer (CC) which includes the two major histology types squamous cellcarcinoma (SCC) and adenocarcinoma (AC), is the fourth most common cancer among women worldwide (3, 15, 28, 29), affecting women under 50 years of age (4) and with approximately 570,000 new cases in 2018 (13.1/100,000 women) (1, 27). Almost all cervical SCCs (CSCC) and some cervical ACs (CAC) are HPV-related and AC is rare compared with SCC (29, 30). Globally, HPV 16 and 18 together account for 71% of cervical cancer, and this percentage rises to 90% for HPV 6/11/16/18/31/33/45/52/58 (4). HPV 16 is the more dominant type in CSCC while HPV18 is more prevalent in CAC (29). In 2018, CC was responsible for 3.3% of deaths due to cancers by causing more than 300,000 deaths, with more than 85% of the deaths occurring in low- to middle-income countries (1). About 98% of CC deaths are attributed to HR-HPVs (1). It is estimated that the highest CC attributable to HR-HPV is in Africa (31.5/100,000 women/year), specifically in sub-Saharan Africa (75.3/100,000 women/year), and lowest in Asia (10.2/100,000 women/year) (1). HR-HPVs are more prevalent in developing countries, mostly due to shortage and/or lack of healthcare access, higher prevalence of immunocompromised patients, a paucity of screening programs, and low vaccination rates (1).

Head and neck squamous cell carcinoma (HNSCC) is the sixth most common malignancy worldwide (7, 31, 32) with 710,000 cases per year (7). HNSCC represents a large and diverse group of malignancies, which have been historically attributed to tobacco and alcohol consumption (3, 4). Although the incidence of HNSCC is declining in some parts of the world, largely due to a decrease in tobacco use, developed countries (*e*.*g*., United States, Canada, Australia, Sweden) have experienced an increase in the incidence of oropharyngeal cancer over the past two decades due to HPV infection, especially in men under 60 years of age (7). HNSCC accounts for about 6% of HPV-attributable cancer (38,000 cases globally), most of which are located in Northern America and Europe (3, 4). HPV 16 and 18 are responsible for 85% of HPV-related cancers of the head and neck (4, 7). Most HPV-related HNSCC arise in the oropharynx (>90%) but has also been detected in other sites, including the oral cavity, larynx, nasopharynx, and sinuses (3, 4).

Although sinonasal malignancies are rare, accounting for approximately 0.2% of all cancers and 3 to 5% of head and neck cancers (5, 33), the sinonasal tract is the second anatomic subsite of the head andneck for HPV-related carcinomas (34, 35). The mean age of patients with sinonasal malignancies is about 62 years, and it is more prevalent in Caucasian men (5). The overall incidence is estimated to be 5 to 9 per million for males and 2 to 5 per million for females based on WHO statistics taken from the GLOBOCAN dataset for 9 countries. Environmental toxins, such as tobacco, and industrial agents, such as wood dust, thorium dioxide, formaldehyde, isopropyl oils, lacquer paints, solder, and welding materials, are risk factors for developing sinonasal malignancies (5, 33). The incidence of sinonasal cancer has been declining in most countries due to decreasing tobacco use and efforts to reduce occupational exposures (5, 33). However, there is increasing acknowledgment that a subset of malignancies isHPV-related but how the virus is transmitted remains unclear (35). HPV type 16 (82%) is themost prevalent, followed by type 31/33 (12%) and type 18 (6%) (34). The most commonsinonasal histologic type is SCC (SNSCC) which accounts for about 60-75% and it is estimatedthat 20% to 62% of SNSCC is HPV positive (36).

## HPV Carcinogenesis

An understanding of transformation processes initiated by HPV infection has relied on the study of premalignant uterine cervical cells and has led to a recognized model of HPV carcinogenesis. The model parallels the normal HPV life cycle with initial infection, establishment, and maintenance, but with persistent infection of basal or stem cells, carcinogenesis can be initiated (37). Persistent infection with HPV, causing genomic instability, is considered a necessary but not sufficient event for the development of cancer (38). There are a variety of molecular mechanisms involved in HPV-associated carcinogenesis that include the overexpression of HPV oncoproteins E6 and E7 altering multiple signaling pathways and inducing genomic instability. Cancer-associated phenotypes are caused by HPV DNA integration in the host genome, immune evasion, changes in global DNA methylation (39–41), and the buildup of genetic and epigenetic modifications or mutations in genes whose encoded proteins act in diverse signaling pathways (42).

The HPV oncoproteins E5, E6, and E7 play a role in infiltrating many signaling pathways to create favorable conditions for cellular transformation. The E5 protein has been demonstrated to play an important role during the productive viral life cycle of HPV (43). The role of E6 and E7 in the initiation and progression of HPV-related cancers has been extensively demonstrated, and together they have been shown to be necessary but not sufficient for HPV-driven cellular transformation (44). E6 targets p53 by forming a complex with the E3 ubiquitin-protein ligase E6-associated protein (E6AP) for proteasomal degradation and can also bind p53 and block transcription of tumor-suppressive genes (39, 41, 45). The degradation of p53 aids in productive viral replication and allows for the accumulation of genetic mutations which can lead to transformation, dysplasia, and cancer (45). Both LR and HR E6 oncoproteins are able to bind to p53, but LR E6 cannot induce degradation (40, 45). HR HPV E7 binds a cell cycle regulator, retinoblastoma protein (Rb), and other retinoblastoma pocket proteins—p105, p107, and p130—for degradation, which results in the release and activation of transcription factor E2F (45). This promotes the expression of S-phase genes, inducing cell proliferation and increased viral gene transcription (45). E7 further induces cell proliferation by promoting the G1–S phase entry of the cell cycle through the inhibition of cyclin-dependent kinase (CDK) inhibitors p21 and p27, leading to the increased activity of CDK2 (41, 45). The degradation of Rb and the increased E2F activity result in a feedback loop, causing an increased expression of the biomarker p16INK4a (p16) which controls the crucial G1–S phase transition (46). LR HPV E7 proteins are still able to target Rb, but with a lower affinity compared to HR HPV E7 proteins, possibly contributing to their difference in progression to cancer (45).

## HPV Attributes, Screening, Diagnosis, Treatment, and Prevention

Almost all cervical cancers are caused by persistent infections with oncogenic strains of HPV, leading to the development of premalignant lesions and, eventually, invasive cancer (40). Since HR-HPV is well established as the main cause of almost all cervical cancers, it has been effectively controlled by screening and diagnosis. Primary screening involves Pap smears that detect morphologic changes in the cervical epithelium (such as abnormal cells and precancerous and cancerous lesions) caused by early HPV infections (30). It is followed by HPV DNA testing if the Pap smear results showed malignancy or co-screening together with HPV DNA testing on the same cytology sample, which gives greater sensitivity and specificity (30). HPV-related cervical cancer histology includes cervical squamous cell (70%), cervical adenocarcinoma (25%), or mixed-histology tumors (30). Non-HPV-related cervical cancer is rare, representing <1% of newly diagnosed cases, with histologies including cervical neuroendocrine, small cell, and large cell carcinomas (30). In comparison of the two major histologies, SCCdevelops from the ectocervix's squamous epithelia and AC develops from the endocervix'sglandular epithelia (29) Studies suggest that the incidence of AC appears to be increasing in some countries while SCC incidence is decreasing (29, 47). The rise is seen among young women, partly due to cohort effect and partly due to cytology screening, which is less effective for detection of AC compared to SCC (29). Although there is growing evidence that ACs have different epidemiology, prognostic variables, patterns of dissemination, and treatment failure following therapy compared to SCCs, both are staged and treated similarly (47). Silvaclassification, which stratifies invasion in three patterns, is used to determine HPV-related CAC (47, 48). Even though p16 expression is considered to be a surrogate marker for HPVassociation, p16 IHC testing is not absolutely necessary for the classification, and HPV analysisis not necessary for the diagnosis (48). HPV-related CSCC causes pre-cancerous lesions but there is no known precancerous lesion in the very rare non-HPV-related CSCC (48). Accordingto WHO guidelines, HPV DNA testing is used to detect HPV-related CSCC but p16 IHC is also recommended since morphology alone cannot distinguish the two types (48). Cervical cancer is a continuous single disease process advancing gradually from mild cervical intraepithelial neoplasia (CIN1) to more severe degrees of neoplasia and micro-invasive lesions (CIN2 or CIN3) and finally to invasive disease (30). The primary treatment for early-stage cervical cancer is surgery and for later-stage type are chemotherapy and/or radiation (37).

HPV-related OPSCC is clinically distinct, affecting younger patients with fewer comorbidities, responding favorably to treatment, and portending survival outcomes compared to HPV-negative OPSCC, affecting older patients with a significant history of tobacco use and alcohol consumption (49, 50). HPV 16 induces over 90% of HPV-related OPSCC, followed by HPV 18 and 45 which presented at less than 2% each (44). Most HPV-related OPSCC present with small primary tumors but often cystic, multilevel nodal disease. The histology is predominantly non-keratinizing SCC with basaloid morphology (9, 51). OPSCC is usually tested for HR-HPV by surrogate marker p16 IHC, and discretionally, additional molecular HPV-DNA testing may also be performed (9, 22). For early-stage OPSCC with minimal or no nodal disease, the treatment is generally either primary surgery and/or definitive radiotherapy (RT) (30). Patients with more advanced disease or the presence of extensive nodal metastases are generally treated with combined modalities, including surgery, radiation, and/or chemoradiation.

While the incidence of sinonasal carcinoma is low, their histology is among the most diverse ofall head and neck sites with several uncommon and distinct subtypes, several SCC variants,interesting etiologic lesions and HPV- related tumors (107). HPV-related sinonasal carcinomahistologic types are SCC and variants (non- or partially-keratinizing, papillary, adenosquamousand basaloid), small cell carcinoma, undifferentiated and carcinoma with adenoid cystic-like features (34), which is now known as HPV-related multiphenotypic sinonasal carcinoma (HMSC) (53–56). HMSC is rare and histologically characterized by multiple patterns of differentiation, including squamoid, ductal, and myoepithelial, similar to adenoid cystic carcinoma (10, 51). There is increasing histologic and epidemiologic evidence suggesting that a subset of SNSCC may be caused by HPV and detection may be a biomarker for improved survival similar to HPV positive OPSCC but definitive conclusions are hampered by small sample sizes and inconsistent HPV detection methods (57). The available literature has shown conflicting results with some studies showing that HPV-related SNSCC is associated with better outcomes,while others have reported that HPV status is not a significant prognostic factor (36). However, HPV testing in these cancers is not widely performed by pathologists. The primary treatment modality is surgery with or without adjuvant RT, with some evidence suggesting that adjuvant RT may prolong the disease-free interval among patients who develop local recurrence (53, 58). **Table 2** shows the summary of a few attributes of HPV-related Cervical cancer, OPSCC and Sinonasal Carcinoma.

**Table 2:** Summary of attributes of HPV-related Cervical cancer, OPSCC and Sinonasal carcinoma.

	Cervical cancer	OPSCC	Sinonasal carcinoma
Histopathology	Keratinizing SCC, AC, large cell nonkeratinizing, small cell nonkeratinizing neuroendocrine	Non-keratinizing SCC with basaloid morphology	Squamoid, ductal myoepithelial non- or partially-keratinizing, papillary, adenosquamous,basaloid, small cell
Molecular diagnosis	HPV-DNA testing p16 immunohistochemistry	p16 immunohistochemistry (and HPV-DNA)	Not recommended
Early-stage primary treatment	Surgery	Surgery and/or RT	Surgery and/or RT
Treatment sensitivity to chemotherapy and radiation	Moderate	High	High

Because almost all cervical cancers and rising proportions of OPSCCs are attributable to HPV infections, universal access to vaccination against HPV could effectively reduce the incidence of these and other HPV-associated cancers (49). By reducing the incidence and transmission of anogenital HPV, the vaccine should also indirectly reduce the incidence and sexual transmission of oral HPV and thereby decrease the incidence of HPV-positive OPSCC (30). Universal HPV vaccination has been introduced into national immunization programs in most developed countries. In Canada, HPV2, HPV4, and HPV9 are available for both sexes from the age of 9 or Grade 6 and are administered as a two-dose series as part of the national immunization program (59). Overall, HPV vaccination has been effective in the prevention of persistent HPV16 and HPV18 infections (39). However, immunization programs are not established in developing countries, and the uptake of the HPV vaccine is low; hence HPV-related diseases continue to rise. HPV vaccination has the potential to prevent almost 90% of cervical and other HPV-related cancers worldwide (30) and will provide the ultimate prevention against HPV-associated diseases among young adults. However, screening and HPV testing will continue to play a key role, as prophylactic vaccines are most effective prior to HPV exposure, and the eradication of HPV through vaccinations is still decades away (30, 60).

## Cervical Cancer Screening and Diagnostic Tools for HPV Detection

Cervical cancer screening and diagnosis is combined liquid-based cytology stained Papanicolaou stain (also known as Pap smear) and HPV testing using DNA/RNA PCR-based methods (12, 13, 61). Papanicolaou carried out the first prospective studies of the vaginal cycle by working with guinea pigs, and in 1943, jointly with Traut, he outlined detailed studies of cycle-dependent epithelial changes in the vaginal epithelium of the human female (62). Epithelial cells are collected from the external surface of the cervix and lower part of the cervical canal using a cervical sampling brush or spatula, processed into a thin layer on a glass microscope slide, stained with Papanicolaou stain, and evaluated by a cytopathologist using a microscope (62). The cytopathologist evaluates the sample by comparing the histologic structure to the normal squamous epithelium from the vagina and ectocervix (62). Höffken et al. (62) summarized the histology and cytology of a normal squamous epithelium from the vagina and ectocervix as shown in **Table 3**.

**Table 3:** Histology and cytology of normal squamous epithelium from the vagina and the ectocervix.

Histology	Cytology	Cytometry C = cell diameter N = nuclear diameter	Proliferation grade
Basal cell layer (stratum basale)	Basal cells, basophilic with dense cytoplasm, nucleus round or oval	C: 12–20 μm N: 8–10 μm	Not seen in normal smears
Parabasal cell layer (stratum spinosum profundum)	Parabasal cells, basophilic with dense cytoplasm, nucleus round or oval	C: 15–25 μm N: 8–10 μm	1
Intermediate cell layer (stratum spinosum superficiale)	Small intermediate cells, polygonal, basophilic, pale-staining cytoplasm, nucleus vesicular, with fine granules	C: 20–40 μm N: 7–9 μm	2
Superficial cell layer (stratum superficiale)	a) Large intermediate cells, polygonal, basophilic, eosinophilic, nucleus still vesicular	C: 40–60 μmN: 6–8 μm	3
	b) Surface cells, polygonal, eosinophilic, basophilic, nucleus pyknotic	C: 40–60 μmN: 6 μm	4

The current reporting system for Pap smears is the Bethesda System, which was introduced in 1988 and amended in 1991 to replace the cervical intraepithelial neoplasia (CIN) system. Burd et al. (13) summarized the cytology and histology terminology for HPV-associated squamous lesions of the cervix, as shown in **Table 4**. The histologic diagnoses are reported as normal, atypia, low-grade squamous intraepithelial lesion (LSIL), high-grade squamous intraepithelial lesion (HSIL), and squamous cell carcinoma (SCC) (12, 13, 63, 64).

**Table 4:** Cytology and histology for HPV-associated squamous lesions of the cervix.

Bethesda system	CIN system	Interpretation
No epithelial abnormalities or benign cellular changes	Normal	Normal
Atypical squamous cells (ASC): ASC-US (undetermined significance), ASC-H (cannot exclude HSIL)		Atypia, squamous cells with abnormalities greater than those attributed to reactive changes but not meeting the criteria for a squamous intraepithelial lesion
Low-grade squamous intraepithelial lesion (LSIL)	CIN 1	Koilocytosis, mild dysplasia, and mild abnormalities caused by HPV infection
High-grade squamous intraepithelial lesion (HSIL). (perform p16 IHC to upgrade or downgrade; if negative, classify as LSIL and if positive, classify as HSIL)	CIN 2-3	Moderate dysplasia, severe dysplasia, carcinoma *in situ*, suspicious; more severe abnormalities that have a higher likelihood of progressing to cancer if left untreated
Squamous cell carcinoma	Invasive squamous cell carcinoma, invasive glandular cell (adeno) carcinoma	Invasive squamous cell carcinoma (cervical cancer) Atypia, glandular epithelial cells

Cytology screening is one of the most successful public health prevention activities worldwide. It has led to significant reductions in cervical cancer incidence and mortality, but it has significant limitations, such as low sensitivity and poor reproducibility (60). HPV testing was more advantageous than cytology largely due to its ability to direct early detection further upstream in cervical carcinogenesis (60). Some of the benefits include the following: (1) higher sensitivity and reproducibility but somewhat lower specificity, (2) ability to be automated, centralized, and be quality-checked for large specimen throughput, (3) more cost-effective than cytology, if deployed for high volume testing, and (4) the ability to use self-sampling, which has the potential to increase screening in remote areas or to women who are not directly reached by primary healthcare in urban areas (60, 65). In 2008, the 3-year prospective study ATHENA (Addressing the Need for Advanced HPV Diagnostics) was initiated in the US, and it is the first and largest screening study to evaluate the performance of HPV primary screening (66). The results indicated that co-testing, cytology, and HPV provided minimal increased protection against the development of CIN2 or worse compared to HPV primary screening. This led the FDA to approve, in 2014, HPV primary screening tests for women ages 25–65. Women tested for HR-HPV 16 and/or 18 are referred for colposcopy, and those positive with the other HR-HPVs should be triaged with cytology; if the latter is positive (ASC-US or worse), colposcopy is recommended. The important development was that the majority of women who tested HPV-negative are to be screened no sooner than 3 years later (60, 61, 66, 67). **Table 5** shows the cervical cancer screening recommendations from the American College of Obstetricians and Gynecologists (ACOG), American Society for Colposcopy and Cervical Pathology (ASCCP), and US Preventive Services Task Force (USPSTF).

**Table 5:** Cervical cancer screening recommendations from ACOG, ASCCP, and USPSTF.

Testing	ACOG	ASCCP	USPSTF
Pap only	Every 3 years	Every 3 years	Every 3 years
Pap–HPV co-test	Every 5 years, age 30–65	Every 5 years, age 30–65	Every 5 years, age 30–65
High-risk HPV only	Every 3 years, age >25	Every 3 years, age >25	Every 5 years, age 30–65

Primary HPV testing followed by cytology was accepted in Canada and Europe because of its safety relative to co-testing and reduction of required tests nearly in half, with a consequent reduction in the cost for screening programs (60). Combining primary HPV screening with cytology triage provides greater reassurance of the absence of cervical lesions and supports increased intervals between screening rounds for up to almost double the maximum duration allowed by conventional cytology (60, 66).

Immunohistochemistry (IHC) for p16INK4a (p16) is commonly used as a surrogate marker for the presence of HR-HPV E7 in tumor tissues and has become the clinical standard for HPV testing (9, 22, 68). Most routine laboratories testing surgical pathologies usually have accessible IHC with pathologists that can easily perform the methods and adequately interpret the staining reactions (69). The IHC assay is widely used in the diagnosis of abnormal cells to identify its origin based on the binding of antibodies (Ab) to specific antigens (Ag) in tissue sections. It is visualized by a histochemical chromogen reaction or by fluorochromes visible by using conventional microscopy or fluorescence microscopy (70). IHC is generally performed on 4–6-μm-thick formalin-fixed, paraffin-embedded (FFPE) tissue slices or on frozen fresh tissue with thickness of 8–90 μm (70). IHC assays detect distinct tissue components by capturing target antigens, with specific antibodies tagged with proper labels binding to the tissues, and the reaction is visualized using fluorochrome (a substance that absorbs or emits light) or chromogens (substances that produce distinct colors) (70). While most pathologists use strong nuclear and cytoplasmic expression for a positive result, a few interpret only cytoplasmic staining as positive (68). The *College of American Pathologists* (CAP) and the *8th edition of the American Joint Commission on Cancer* (AJCC8) recommend that, for a result to be considered positive, a threshold of at least 70–75% of tumor cells must show moderate to strong nuclear and cytoplasmic staining of the neoplastic cells. The threshold of at least 70% of positive tumor cells might be too high because it was found that there is a presence of nuclear and cytoplasmic staining in 50–70% of tumor cells associated with HR-HPV in a subset of patients (71). IHC for the detection of p16 expression is a highly sensitive surrogate marker for transcriptionally active HR HPV infection in CSCC (in the triage of women with positive screening results and to identify pre-cancer biopsies) (72).

*In situ* hybridization (ISH) is a method used to detect nucleotide sequences based on the complementary binding of a nucleotide probe (cDNA, cRNA, or synthetic oligonucleotide) to a specific target sequence of RNA or DNA in cells, tissue sections, or an entire tissue (73). The hybrids that form between the labeled probe and the specific target sequences can be visualized and detected by various methods (73). Tissue samples are prepared by the treatment with proteases to facilitate access of the target nucleic acid to increase hybridization efficiency and reduce nonspecific background staining (73). The probes used have radioisotope labels or non-isotope labels (biotin, fluorescein, digoxigenin, alkaline phosphatase, or bromodeoxyuridine are used) (73). Radioisotope labeling is considered as the most sensitive, but others believe that nonisotopic methods are just as sensitive (73). The radioisotope labeling hybridization sites are observed by autoradiography with an X-ray film or liquid emulsion, and the nonisotopic labeling hybridization sites are observed by histochemistry or immunohistochemistry (73). The HPV detection procedure in ISH occurs within the nuclei of infected cells, which makes it the only molecular method that reliably detects and identifies the location of specific nucleic acid sequences in tissues, which is evaluated microscopically (74). The presence of HPV in tissue samples being tested is indicated by the development of appropriate precipitate within the nuclei of the epithelial cells, and the condition of the virus can be classified as integrated or episomal by the presence of punctuating signals and diffuse signals, respectively (74). ISH is highly specific (100%) but not sensitive (83%) for HPV infection compared with p16 immunohistochemical staining (73, 74).

Polymerase chain reaction (PCR) is a widely used technique that allows a specific stretch of DNA to be copied exponentially in a short amount of time (75–77). There are five primary components of PCR, and it is summarized in **Table 6**. They are as follows: (1) template DNA, the double-stranded DNA segment to be copied; (2) deoxynucleotide triphosphates (dNTPs), the building blocks of DNA [adenine triphosphate (ATP), thymine triphosphate (TTP), guanine triphosphate (GTP), and cytosine triphosphate (CTP)]; (3) polymerase enzyme, Taq DNA polymerase joins the nucleotides together; (4) oligonucleotide primers, DNA sequence complementary to the target DNA; and (5) buffer solution of favorable ionic strength and pH (75).

**Table 6:** Summary of PCR components and description.

Component	Description
Template DNA	Double-stranded DNA segment to be copied
dNTPs	The building blocks of DNA. The 4 nucleotides are ATP, TTP, GTP, and CTP
Polymerase enzyme	Taq DNA polymerase enzyme which joins the nucleotides together, creating a mirror image of the template
Oligonucleotide primers	DNA sequence complementary to the target DNA where DNA polymerase binds and initiates DNA synthesis
Buffer solution	A solution to contain the DNA sample of favorable ionic strength and pH

PCR uses Taq DNA polymerase derived from the thermophilic bacterium *Thermus aquaticus* for its heat stability, as it allows the enzyme to withstand the heating needed to denature DNA and maintain activity at relatively high temperatures which improve primer specificity (75). There are three core steps involved in PCR, as summarized in **Table 7**—step 1: denaturation is heating the PCR tube components at high temperatures (94–96°C), which weakens the DNA and breaks the two complementary strands apart; step 2: annealing is cooling the PCR tube components (55°C), which allows the DNA primers to bind themselves to the complementary sites on the template strands; and step 3: extension is heating the PCR tube components (72°C), which permits the DNA polymerase to copy the template strands by adding nucleotides onto the ends of the primers and producing two molecules of double-stranded DNA (75). The process is normally repeated through a number of cycles, thereby increasing the amount of the target DNA exponentially (75).

**Table 7:** Summary of the steps and events in PCR.

Steps	Event
Denaturation	A very small PCR tube is heated to 94–96°C, which denatures the DNA and splits the two complementary strands apart
Annealing	The tube is cooled, which allows the DNA primers to bind themselves to the complementary sites on the template strands
Extension	The DNA polymerase copies the template strands by adding nucleotides onto the ends of the primers and producing two molecules of double-stranded DNA

PCR is an integral component of many protocols and is perhaps the key technique of molecular biology (75). PCR has broad applications, including medical diagnostics, and as such, it is used to detect HPV. PCR is a selective technique capable to reproduce and increase the amount of target HPV sequences present in biological specimens exponentially, following repeated cycles of amplification (77). PCR-based assays have wide-ranging specificity and sensitivity determined by a few factors such as the size of the PCR product, the spectrum of HPV DNA amplified and ability to detect multiple types, the primer sets chosen, the reaction conditions, and the performance of the polymerase enzymes in the reaction (77). Most primer sets are designed to target the L1 gene or the E6 and E7 oncogenes (78). PCR primers directed at the E6 or E7 regions have been described as preferable because the L1/E1 regions are often lost during the integration of viral DNA into host genomic DNA, and targeting the L1 or E1 region may miss advanced disease (77).

The most current HPV detection methods that are commercially available are type-specific target amplification DNA PCR and signal amplification DNA ISH, which are approved for cervical samples (77, 78). HPV DNA PCR is a target amplification technique that effectively amplifies small amounts of DNA sequences in a biological specimen containing diverse cell types, using primers that can be specific for a single HPV type or target sequence shared by multiple types (78). HPV DNA PCR can also be used as a non-quantitative technique, but information about the abundance of a particular DNA species is not provided (78). DNA ISH is a signal amplification technique that utilizes labeled DNA probes (that can be type specific to one HPV type or multiple HPV types or mixed in a single reaction to cover a range of HPV types) that bind to a specific target sequence of DNA-forming hybrids visualized using microscopy (73, 78). The performance of DNA PCR and DNA ISH is comparable, but a direct comparison suggests that DNA ISH may be more practical as a diagnostic tool due to its ability to reliably differentiate relevant HPV infection from passenger virus or contaminant (78). Furthermore, DNA ISH adaptation to FFPE tissues makes it compatible with standard tissue processing procedures, using nonfluorescent chromogens that allow hybridized DNA to be visualized using conventional light microscopy and the introduction of various signal amplification steps that has increased sensitivity (78).

Hybrid Capture 2 (HC2) HPV DNA test was developed by Digene Corporation (Gaithersburg, MD) and is now marketed by Qiagen (Germantown, MD) and approved by the FDA in 1999, and it replaced the original Hybrid Capture (HC1) tube-format assay, which was approved in 1995. It was the only test available until 2009. The HC2 is a microtiter-format nucleic acid hybridization assay with signal amplification for cervical specimens collected using the HC2 DNA collection device or HC cervical sampler (cervical broom) (13). The specimen release and denature target DNA after treatment, and a mixture of multigene RNA probes specific for 13 high-risk HPV—16, 18, 31, 33, 35, 39, 45, 51, 52, 56, 58, 59, and 68—is added (13). If HR HPV is present, it combines with the probes, and the resultant DNA–RNA hybrids are captured onto the wells of a microtiter plate that are coated with monoclonal antibodies to DNA–RNA hybrids (13). The addition of a second monoclonal antibody conjugated to the alkaline phosphatase binds to the captured hybrids in multiples, resulting in dephosphorylation of a chemiluminescent substrate which produces light (13). The alkaline phosphatase acts on many copies of the substrate, creating an amplified target/signal level, and the emitted light is measured in relative light units (RLU) on a luminometer (13). HR probe may cross-react with LR HPV that is not in the probe mixture, which will adversely affect the sensitivity (77, 79). The HC2 test has a cutoff of 1 RLU, and an RLU greater than or equal to 1 indicates the presence of HR HPV DNA, while an RLU less than 1 indicates either the absence of HR HPV DNA or HR HPV DNA levels below the limit of detection of the test (13). The test has a sensitivity of 0.2 to 1 pg/ml, which is equivalent to 1,000 to 5,000 genome copies of HPV, but does not distinguish the specific HPV genotype present (13, 79). It is not possible to determine the quality of the specimen or the presence of potentially interfering substances because HC2 test does not contain an internal control (13, 79).

The Cervista HR HPV test (Third Wave Technologies, Madison, WI, USA; now Hologic/Gen-Probe, San Diego, CA) was approved by the FDA in 2009. It utilizes proprietary Invader Chemistry to generate signal amplification of a fluorescent probe to detect HPV DNAs from 14 HR types, including the same 13 types detected by the HC2 test plus HPV66 (13, 79). The analytical sensitivity of the Cervista HPV HR test varies depending on HPV type, with limits of detection of 1,250 to 2,500 copies per reaction for HPV16, 18, 31, 45, 52, and 56, 2,500 to 5,000 copies per reaction for HPV33, 39, 51, 58, 59, 66, and 68, and 5,000 to 7,500 copies per reaction for HPV35 (13, 79). Similar to HC2, it does not identify the individual HPV type (13, 79). Cervista uses a lower sample requirement of 2 ml (*vs*. 4 ml) and has lower cross-reactivity with some LR HPV types compared to HC2 (13, 79). Its analytical sensitivity is comparable to HC2, but it uses the human histone 2 gene as an internal control to ensure the efficacy of the specimen and eliminate false-negative results (80).

The APTIMA HPV assay (Hologic Gen-Probe Inc., San Diego, CA, USA) was approved by the FDA in late 2012. The assay qualitatively detects E6/E7 mRNA transcripts of 14 high-risk HPV types and uses a noninfectious RNA transcript as extrinsic process control (13). The assay performs pooled HR HPV detection that does not distinguish between the 14 targeted HR types like HC2 and Cervista HR HPV assays. The 3 main steps in the assay, which occur in the same tube, involve target capture, target amplification using transcription-mediated amplification, and detection (79, 81). The assay uses 1 ml of liquid-based cytology, and a lesser amount is inadequate for testing (79, 81). The cells are lysed so that mRNA can be released and allowed to hybridize to capture oligonucleotides attached to magnetic microparticles. (79, 81) The bound target mRNA moves to the side of the tube by the utilization of magnetic fields, and the supernatant is aspirated and then washed (79, 81). The captured HR HPV mRNA is amplified by transcription-mediated amplification, detected by hybridization protection assay using chemiluminescent labels (13, 79). A luminometer is used to measure the resultant signal in RLUs, and the results are interpreted based on the analyte signal-to-cutoff (S/CO) value (79). Internal control (IC) is added to each reaction, and the signal in each reaction is distinguished from the HPV signal by the differential kinetics of light emission from probes with different labels (79). Target RNA amplification is detected using probes with a slow emission of light (glowers), and IC amplification is detected using probes with a rapid emission of light (flashers) (79). The dual kinetic assay is a method used to differentiate between the signals from the flasher and glower labels (79). The analyte S/CO is calculated from the analyte RLU of the test sample and the analyte cutoff for the run (79). If the S/CO ratio is <0.50, a negative result is generated, and if the S/CO ratio is ≥0.50, a positive result is generated (79). The system is automated with high output, and the full process from sample preparation to result detection can be automated on the TIGRIS system (Hologic) (13, 79).

The Cobas 4800 HPV test (Roche, Pleasanton, CA, USA) was approved by the FDA in 2011 but has been available in the European market since 2009. It is a target amplification assay that detects the same 14 HR HPV types as the Cervista and APTIMA tests but also distinguishes HR-HPV types 16 and 18 (13, 79, 80). It simultaneously detects the L1 gene of HPV16 and HPV18 as individual reactions and the other 12 HR-HPV as a pooled result by using multiplex real-time PCR and nucleic acid hybridization with four different fluorescent reporter probes (13, 79). There are four fluorescent-labeled cleavage primer probes used for detection of amplification of the HPV DNA that target the L1 region: one specific for HPV 16, one specific for HPV 18, one for non-16/18 genotypes, and one for β-globin (79). The test is automated, and the system consists of two separate instruments: the Cobas z 480 instruments for automated nucleic acid extraction and the Cobas x 480 analyzers for PCR amplification and detection reactions in a single tube (13). The system is designed to process up to 280 cervical specimens collected in PreservCyt solution in 1 day (13). False negatives can occur though since the L1 gene is lost upon integration into the human genome in a considerable proportion of cancers (13, 79). The overall intra-laboratory agreement is 98.3%, and genotyping agreement is 98.2%. Inter-laboratory reproducibility studies showed 94.6% overall agreement and 93.7% genotyping agreement (79).

The OncoE6^™^ Cervical Test (Arbor Vita Corporation, Fremont, CA) is a qualitative lateral flow assay (strip test) that detects the elevated level of E6 oncoprotein expressed from HPV infected cells associated with the most common oncogenic HPV types 16 and 18 (82, 83). The presence of elevated E6 oncoprotein levels suggests that there is an existing malignant cell or an increased risk of future malignancy (82, 83). The assay uses cell lysates samples from cervical swab specimens or from specimens collected in PreservCyt® solution (82). The lysate is incubated with highly specific mouse monoclonal antibodies (mAbs) to E6 oncoprotein from HR-HPV types 16 and 18 bound with alkaline phosphatase (AP) (82). The test strip made out of nitrocellulose with two capture lines consisting of the immobilized mAbs to E6 16/18 is placed in the lysate/mAb-AP mix (82). By capillary action, the lysate/mAb-AP mix pass through the test strip, and a complex (capture mAb-E6-detector mAb) may form if E6 16 and/or 18 is present and becomes visible as a purple line at the respective locations (either 16 or 18) when the enzyme substrate is added (82). If the test is valid and a purple test line at any intensity is seen, the result is positive and no line indicates a negative result (82). The assay was validated in several clinical studies. Valdez et al. (84) conducted cervical cancer screening study in rural China and their results showed that OncoE6TM Test had a 70.3% sensitivity and 98.9% specificity for CIN3 detection compared to HPV DNA testing (careHPV) and visual inspection with acetic acid (VIA). Torres et al. (85) performed a cervical cancer screening in remote areas in Brazil and their results showed that OncoE6^™^ has overall 50% sensitivity and 99% specificity for CIN3+ and specificity is a high priority in remote geographic settings due to the difficulties of follow up. Krings et al. (83) demonstrated that OncoE6^™^ has a high sensitivity in the detection of HPV 16or 18 in 3 different types of self-sampled specimens and their results showed 90% sensitivity with the Delphi Screener lavage and the cytobrush sample in PreservCyt media and 95% sensitivity for the swab sample. They suggested that using OncoE6^™^ testing and self-sampled specimens will allow highly effective cervical cancer screening in remote areas, thereby increasing the effectiveness of preventive strategies (83). The comparison of the diagnostic tools of CSCC is summarized in **Table 8**. In testing women with abnormal cytology, HPV testing is more sensitive (97.4 *vs*. 56.4%) and more reproducible (Cohen’s kappa coefficient *k* = 0.60–0.93 *vs*. *k* = 0.46) but less specific (94.3 and 97.3%) compared to cytology for the detection of cervical pathology (13, 79). For the detection of CIN2 + in women with abnormal cytology, p16 IHC sensitivity compared to cytology is 85.7 *vs*. 54.7% and for specificity 88 and 61%, respectively (79). All of the FDA-approved assays for HPV detection use either target or signal amplification techniques and are approved for use with liquid-based cytology. For the sensitivity comparison of HC2, APTIMA, and Cobas 4800 (96.3, 95.3, and 95.2%, respectively), HC2 is most sensitive, and for specificity (19.5, 28.8, and 24.0%), APTIMA is more specific (79). The HC2, Cervista, and Cobas 4800 tests target HPV DNA, while the APTIMA tests target E6/E7 mRNA and have improved specificity compared to the other assays. They all have similar sensitivity for the detection of cervical dysplasia (79).

**Table 8:** Summary of performances of the tests for CSCC.

Test	Sensitivity, %	Specificity, %	Reproducibility
Cytology	53.3	92	
p16 IHC	85.7 (88^a^)	54.7 (61^a^)	
OncoE6^™^	50-70	99	
Cytology	53.3	92	*k* = 0.46
HPV testing	73.0	56.9	*k* = 0.60–0.93
HPV testing methods			
HC2	96.3	19.5	
APTIMA	95.3	28.8	
Cobas 4800	95.2	24.0	
OncoE6TM	50-70	99	

a If performing p16 on HPV-positive women only.

## OPSCC Screening and Diagnostic Tools for HPV Detection

The current recommendation for HPV testing for OPSCC from the *College of American Pathologists* (CAP) and A*merican Society of Clinical Oncology Guidelines* (ASCO) is p16 IHC, and additional molecular HPV-DNA testing may also be performed at the physician’s discretion. However, HPV testing is not recommended for other HNSCC (9, 21, 22). There is evidence that p16 IHC shows strong diffuse cytoplasmic and nuclear staining in >70% of the tumor cells in SNSCC, though a lower rate than that for OPSCC (86) can be used as a surrogate marker (21, 34, 86). Since SNSCC is not studied as much as OPSCC due to its rarity, the favorable effect of HPV diagnosis is inconclusive and therefore p16 IHC testing is not a routine practice (52, 87). Future research studies are essential to better understand the role of HR HPVs in sinonasal carcinoma. p16 IHC is currently used as a highly sensitive surrogate marker for detecting transcriptionally active HPV in OPSCC (both primary and metastatic sites) (21). Other HPV testing methods are also utilized, such as viral DNA detection by PCR or ISH; the combined detection of p16INK4a IHC and HPV DNA-PCR is frequently applied as well (68). The E6 oncoprotein testing has also been used to detect HPV in HNSCC. Menegaldo et al. (88) detected HPV16/18 E6 oncoproteins in 34 OPSCC and (cancer of unknown Primary) CUP usingOncoE6TM and their results showed 94% and 88% sensitivity when applied to the primary tumorand neck nodes respectively and 100% specificity in both primary and neck lesions. Cherneskyet al. (89) evaluated HPV E6 oncoproteins and nucleic acids in FNA and oral samples frompatients with OPSCC using commercial assays. Their results showed that for FNA samples, theoverall agreements of p16 antigen staining of tumor were 81.4% (k 0.53) for OncoE6™, 94.9%(k 0.83) for Aptima HPV E6/E7 mRNA and 91.1% (k 0.73) for cobas HPV DNA (89). Therewere lower agreements with tumor markers for saliva and oral swab samples; 23.7–24.0% (k0.02) for OncoE6™, 55.9–68.0% (k 0.24–0.37) ) for Aptima HPV E6/E7 mRNA and 78.9–86.9% (k 0.49–0.58) for cobas HPV DNA (89). The E6 oncoprotein testing has also been used to detect HPV in HNSCC. Menegaldo et al. (88) detected HPV16/18 E6 oncoproteins in 34 OPSCC and (cancer of unknown Primary) CUP using OncoE6TM and their results showed 94% and 88% sensitivity when applied to the primary tumor and neck nodes respectively and 100% specificity in both primary and neck lesions. Chernesky et al. (89) evaluated HPV E6 oncoproteins and nucleic acids in FNA and oral samples from patients with OPSCC using commercial assays. Their results showed that for FNA samples, the overall agreements of p16 antigen staining of tumor were 81.4% (k 0.53) for OncoE6™, 94.9% (k 0.83) for Aptima HPV E6/E7 mRNA and 91.1% (k 0.73) for cobas HPV DNA (89). There were lower agreements with tumor markers for saliva and oral swab samples; 23.7–24.0% (k 0.02) for OncoE6^™^, 55.9–68.0% (k 0.24–0.37) ) for Aptima HPV E6/E7 mRNA and 78.9–86.9% (k 0.49–0.58) for cobas HPV DNA (89). Agustin et al. (71) summarized the benefits and drawbacks of HPV detection techniques for OPSCC, as shown in **Table 9** with the addition of OncoE6^™^ testing. p16 IHC sensitivity in OPSCC is around 80–90%, and specificity varies from 80 to 90% (71). p16 IHC is a cost-effective method, and its diagnostic performance is considered high enough to diagnose HR HPV infection in OPSCC (71). DNA PCR techniques are known to be stable and reproducible and have a sensitivity of 98% and specificity of 84% (68, 71). RT PCR detection of HPV mRNA E6/E7 has a sensitivity of 97% and specificity of 100% and is considered by some authors to be the gold standard to diagnose HPV-related OPSCC, but it requires fresh/frozen specimens and is technically demanding and therefore not useful for routine screening (68, 71). HPV DNA ISH allows for direct visualization of the virus within the tumor cells and minimizes the risk for a false-positive test result that may derive from tissue contamination with viral DNA. HPV DNA ISH has a sensitivity of 85% and specificity of 88% (68).

**Table 9:** Summary of HPV detection techniques used in OPSCC.

Detection method	Advantages	Disadvantages	Sensitivity, %	Specificity, %
p16 IHC	High sensitivity Inexpensive FFPE tissues manageable	Moderate specificity	80–90	80–90
DNA PCR	HPV genotype information High sensitivity FFPE tissues manageable Easy and inexpensive	No information about viral transcription High risk of contamination (intrinsic and extrinsic)	98	84
E6/E7 mRNA RT-PCR	High sensitivity and specificity Detects active viral infection Gold standard for research	Time-consuming Non-FFPE tissues manageable (fresh or frozen tissue only) RNA fragility RNA degradation over time, expensive	97	100
E6/E7 mRNA ISH	High specificity and sensitivity *In situ* detection of a transcriptionally active HPV infection FFPE tissues manageable	RNA degradation over time Expensive	87–100	88–100
HPV DNA ISH	*In situ* detection of HPV DNA High specificity FFPE tissues manageable	Low sensitivity	85	88
OncoE6^™^	High specificity, easy to use	Low sensitivity, only for HPV 16 and 18, needs to be validated with a larger cohort	88-94	100
Serology for antibodies against E6 protein	Present in more than 90% of patients with OPSCC related to HPV16 Easy to set up	Lack of clinical data and retrospective		
HPV circulating tumoral DNA by ddPCR	Early detection of recurrences in post treatment monitoring High sensitivity and specificity, low cost	Needs to be validated		

New HPV biomarkers have been studied in the management of HPV-related OPSCC. Antibodies against E6 protein have been associated with a 132-fold increased risk in developing OPSCC and develop more than 10 years before HPV-related OPSCC diagnosis (71). Research showed that these E6 antibodies are detectable in less than 1% of healthy individuals, but other studies have shown that most HPV-positive OPSCC patients (>90%) present an HPV16 E6 antibody response in blood at the time of their HPV16-OPSCC diagnosis (71). Some researchers suggest that E6 serology could be considered for HPV OPSCC monitoring, especially in tracking a residual disease or recurrence, but more validation and research is needed before consideration for clinical routine application (71).

The detection of HPV circulating tumoral DNA (ctDNA) from plasma by using ultra-sensitive droplet digital PCR (ddPCR) has garnered a growing clinical interest in HNSCC and CSCC. HPV ctDNA detection in the plasma of HPV-related OPSCC patients using ddPCR is highly sensitive and specific in identifying HPV16 and HPV33 subtypes in a similar distribution as reported in major genomic profiling studies (90). Their results suggested that HPV16 and HPV33 ctDNA ddPCR could be used in early detection screening trials and in disease response monitoring. The HPV ctDNA in CSCC detection using ddPCR may predict relapse, and their results suggest that monitoring HPV ctDNA could help evaluate treatment options for patients with residual HPV ctDNA after treatment (91). ddPCR and RT-PCR performances were compared in the detection of HPV ctDNA in cervical neoplasia at different stages of the disease, and ddPCR offers sensitive detection and absolute quantification of low target DNA compared to RT-PCR (92).

The quantitative method of ddPCR is characterized by its high sensitivity, its accuracy, and its inter-laboratory and intra-laboratory reproducibility (31, 71). The ultrasensitive ddPCR can be operated at a very low cost compared to other innovative technologies (71). These properties of ddPCR can be applied to detect samples in swabs with very low amounts of DNA.

## Droplet Digital Polymerase Chain Reaction for HPV Detection

ddPCR quantifies the absolute amount of target nucleic acid molecules encapsulated in discrete, volumetrically defined water-in-oil droplet partitions (23, 93). It was first commercially available in 2011 (94), but the concept of ddPCR was first raised by Sykes in 1992, in which DNA molecules are quantified using Poisson distribution and diluting templates to single-molecule level (95). The samples are prepared in a similar manner as the PCR reactions that use TaqMan hydrolysis probes or DNA binding dyes (Eva Green^®^) but in smaller volume-precise reactions or partitions which are then run individually. Positive reactions are checked and calculated among each partition using Poisson distribution (95, 96). The system involves 3 main parts as follows (also summarized in **Table 10**): (1) droplet generation, in which the samples are placed in a droplet generator to partition each sample into 20,000 uniform, nanoliter-sized droplets, enabling precise target amplification; (2) amplification, in which samples are placed in a thermal cycler to amplify each droplet, following the PCR principle involving denaturation, annealing, and extension; and (3) droplet reading, in which the droplet reader reads spaced-out individual droplet fluorescence in two channels (93).

**Table 10:** Summary of the steps and events in ddPCR.

Steps	Events
Droplet generation	The samples are placed in a droplet generator using specially developed reagents and microfluidics to partition each sample into 20,000 uniform, nanoliter-sized droplets, enabling precise target quantification. The target and background DNA are distributed randomly into the droplets. **Figure 1** shows the partitioning of discrete droplets and the distribution of target and background DNA (93)
Droplet amplification	The droplets are transferred in a thermal cycler to amplify each droplet. The amplification of target molecules follows a similar principle of RT-PCR which involves denaturation, annealing, and extension (93)
Droplet reading	The droplets are streamed in a single file in the reader which calculates the target DNA concentration by counting the fluorescent positive and negative droplets in two channels. The positive droplets containing at least one copy of the target DNA molecule demonstrate increased fluorescence compared to negative droplets. **Figure 2** shows the separation of individual droplets and readings measured in two channels (93)

**Figure 1:**
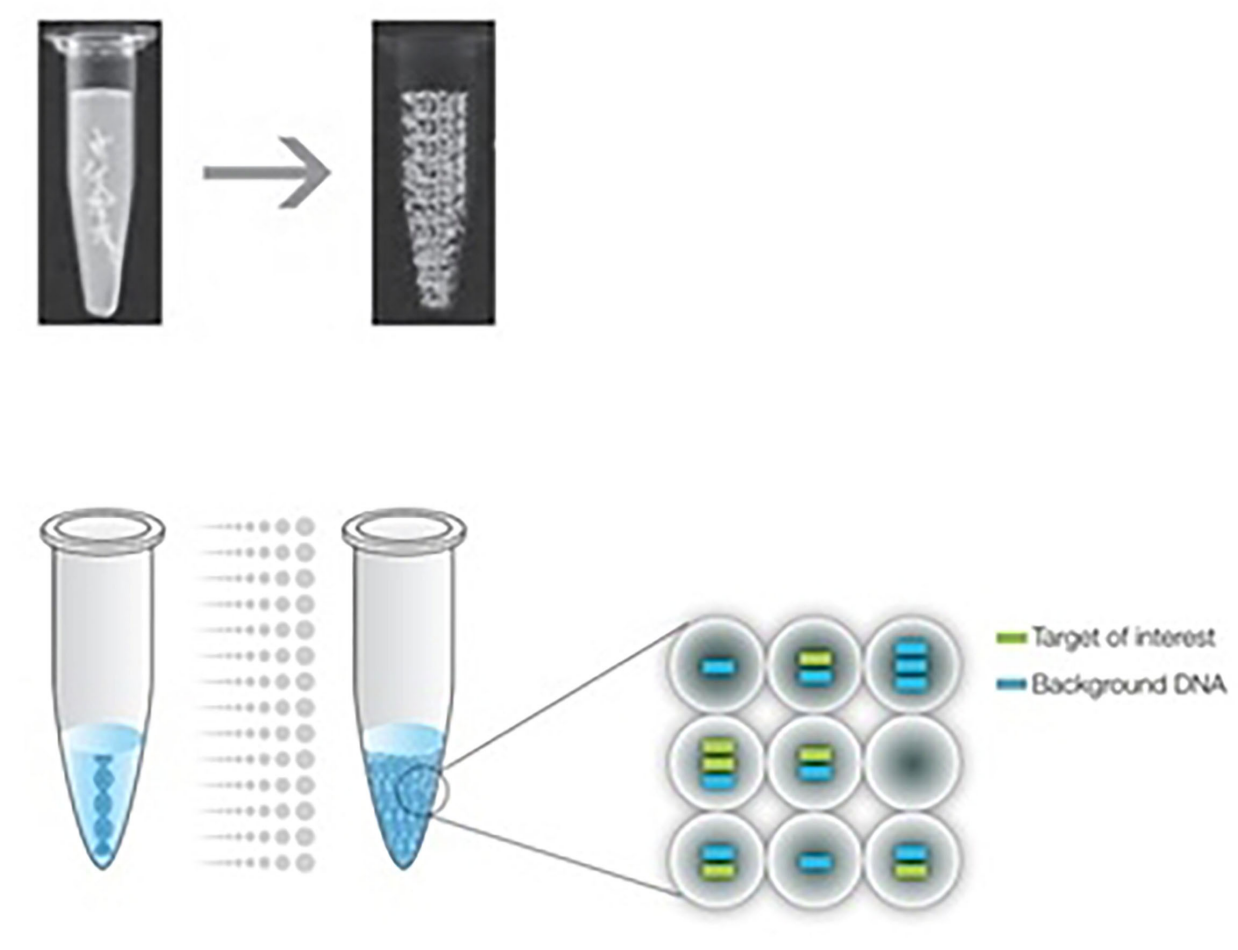
The ddPCR sample is partitioned into 20,000 uniform, nanoliter-sized droplets, and the target and background DNA are distributed randomly into the droplets. (93).

**Figure 2:**
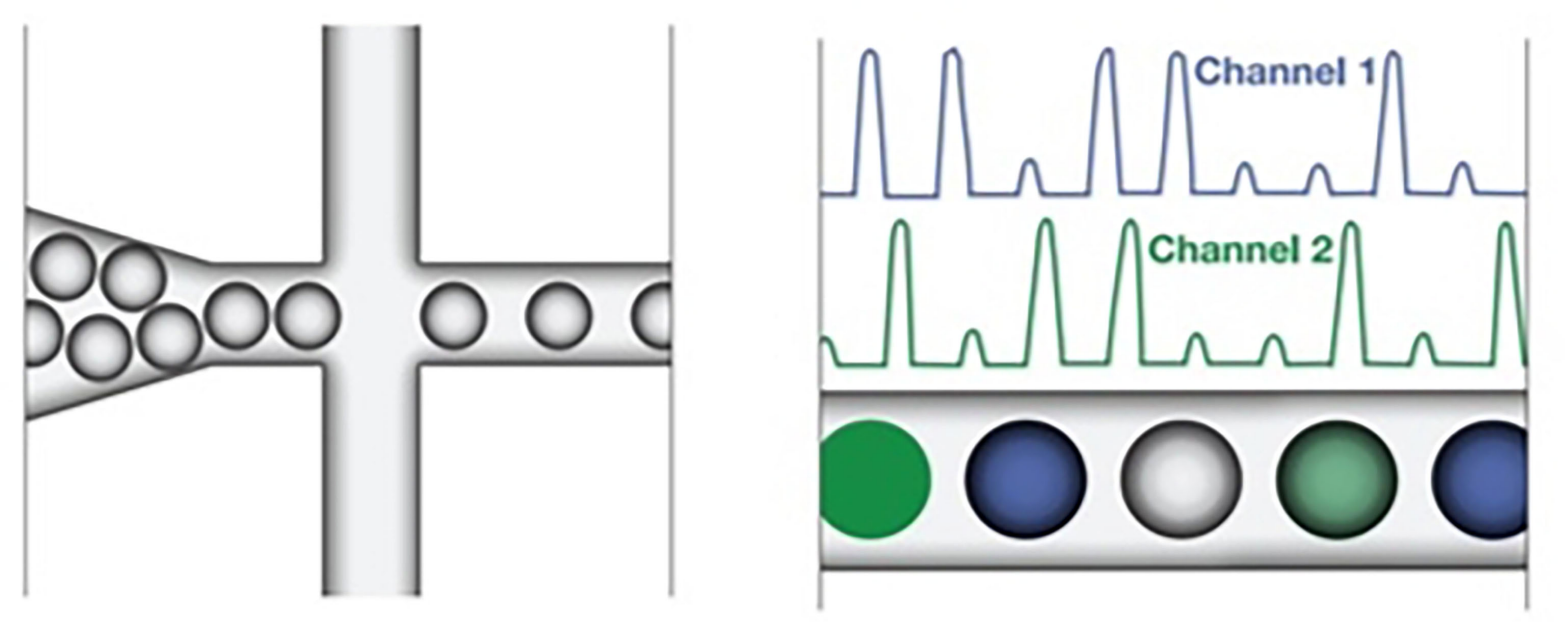
The droplet reader reads spaced-out individual droplet fluorescence in two channels in positive droplets with at least one copy of the target DNA molecule demonstrating increased fluorescence compared to negative droplets. (93).

ddPCR has a broad range of applications, as summarized in **Table 11**, in both research and clinical diagnostic applications, such as (1) absolute quantification for target DNA measurements, viral load analysis, and microbial quantification, (2) genomic alterations such as gene copy number variations (CNV), (3) detection of rare sequences, (4) gene expression and microRNA analysis, (5) next-generation sequencing, (6) single-cell analysis, and (7) genome edit detection (93).

**Table 11:** Summary of the applications and capabilities of ddPCR.

Applications	ddPCR capabilities
Absolute quantification	ddPCR’s immense droplet partitioning provides quantification of DNA copies without standard curves, giving more precise and reproducible data and making it ideal for target DNA measurements, viral load analysis, and microbial quantification (93)
Genomic alterations such as gene copy number variation (CNV)	CNVs are deletions and amplifications of genome segments involved in phenotypic variability, complex behavioral traits, and disease. ddPCR’s droplet partitioning provides a large number of replicates that precisely measure copy numbers (23, 93)
Detection of rare sequences	ddPCR increases sensitivity by partitioning the target mutant DNA away from highly homologous wild-type DNA (93)
Gene expression and microRNA analysis	ddPCR provides stand-alone absolute quantification withsensitivity and precision of expression levels, especially low-abundance microRNAs (93)
Next-generation sequencing (NGS)	Absolute quantification and accuracy of NGS sample preparations and validated sequencing results or CNVs (93)
Single-cell analysis	ddPCR enables the quantification of low copy number (93)
Genome edit detection	dPCR provides fast, accurate, and cost-effective evaluation of homology-directed repair and non-homologous end joining generated by CRISPR-Cas9 or other genome editing tools (93)

### ddPCR HPV Detection in CSCC and OPSCC

The high sensitivity, specificity, and absolute quantification for target DNA measurement by ddPCR are particularly of interest for HPV detection. Several studies have used ddPCR to detect HPV DNA and viral load (VL) in CSCC. HPV VL is an important determinant of virus persistence, and therefore VL quantification is a useful tool in preventive strategies as well as a biomarker for monitoring treatment response and prognosis estimation in HPV-related diseases (96, 97). ddPCR was used to detect HPV in CSCC by using FFPE tissues, cervical liquid cytology samples, and cell lines. Malin et al. (96) detected HPV VL in FFPE tissues and cervical liquid cytology, and their results showed that ddPCR was highly sensitive in detecting HPV and VL at the lowest dilution level, there was no difference in VL between tumors with multiple and single HPV infections and women’s age, and HPV genotype and genera were associated with VL (96). Larsson et al. (97) compared ddPCR with qRT-PCR in quantifying HPV VL in FFPE tissues and liquid-based cytology (LBC). Their results showed that DNAs extracted from FFPE tissue samples yielded lower amplification signals compared to LBC samples, and ddPCR was found to quantify copy numbers that are 1 to 31 times higher than qRT-PCR numbers (97). Rotondo et al. (27) used ddPCR to quantify HPV DNA in CIN specimens and human cell lines, and their results showed the reliability of ddPCR in the simultaneous detection and quantification of different HPV types in one experimental run and low-template-copy-number conditions (27). ddPCR exhibited high sensitivity, accuracy, and specificity in quantifying HPV DNA sequences, and the method was repeatable and reproducible (27).

HPV detection using ddPCR has been demonstrated in OPSCC FFPE tissues, tissue biopsy, fine needle aspiration (FNA) biopsy, and swabs. Schiavetto et al. (98) detected HPV DNA in OPSCC FFPE tissues and showed comparable results to the clinical standard technique p16 IHC (98). Antonsson et al. (99) detected HPV 16 VL in OPSCC FFPE tissues and showed large variations among HPV 16-positive OPSCC ranging from 1 copy per cell to over 900 per cell compared to CSCC where high VL is associated with an increased risk of CIN progression (99). Biron et al. (26) detected HPV 16 in OPSCC tissues, FNA, and swabs, and they showed that adequate amounts of RNA were extracted using commercially available kits, and the sensitivity and specificity of HPV E6 and E7 ddPCR for the detection of p16 positivity was 91.3 and 100%, respectively, compared against p16 IHC (26). Isaac et al. (25) detected HPV 16 in OPSCC swabs showing 92% sensitivity and 98% specificity against fresh tissue p16 IHC, which is the clinical reference standard (25). The excellent sensitivity and specificity of HPV detection using ddPCR in swabs without the need for invasive tissue biopsy have several potential applications for both diagnosis and disease surveillance. Furthermore, the ddPCR method is reported to be accurate, repeatable, reproducible (27, 94, 100), and cost-effective (23, 25, 26, 90).

### ddPCR for Cervical HPV Self-Sampling

Several studies have demonstrated the effectiveness of self-sampling vaginal swabs as a screening tool for CSCC in the minorities and lower socioeconomic groups, remote or hard-to-reach areas, and low-resource settings. The HPV self-sampling was effective in detecting HPV and as sensitive as clinician cytology samples to detect CIN2 or higher (15, 16, 101–103). The study of Wright et al. (18) found that HPV testing of the self-sampled vaginal swab is less specific but as sensitive as cytology for detecting high-grade cervical disease in women age 35 years and older, while the study of Sancho-Garnier et al. (103) found that the sensitivity and specificity of HR-HPV testing using self-sampled vaginal swabs is very similar to that of clinician-collected cervical specimens. Gustavsson et al. (104) showed that self-sampling and repeated HPV tests detected more than twice as many women with CIN2+ compared to Pap smear cytology. Irregular or absenteeism to cervical screening is a major barrier to eliminating cervical cancer, and there are many reasons for low participation, such as cultural reluctance (14, 16, 104), limited access to healthcare or geographical isolation (105), lack of health insurance, low health literacy, language barriers, and lack of awareness (16). HPV self-sampling is a great tool to increase cervical screening, and several studies reported high uptake in participation (14, 16, 101, 105–108). Moses et al. (107) reported that there was a high uptake of self-sampling HR-HPV testing, and it was highly acceptable in the community for cervical cancer screening which exceeded 99%, whereas the standard of care, visual inspection with acetic acid, reached only 48.4% in a low-resource setting. Women have positive experiences and a highly accepted HPV self-sampling screening strategy (14, 15, 106). Furthermore, in a randomized trial performed by Haguenoer et al. (108), they showed that HPV self-sampling is a cost-effective method to increase participation in a cervical cancer screening program. With the substantial amount of studies performed on HPV testing of self-sampled specimens with positive outcomes, it has been proposed to be considered as a screening tool (14, 15, 101, 105, 107, 108). Self-sampling at home followed by HR-HPV testing has been proposed to increase screening recruitment among underserved groups for convenience and to avoid the need for a gynecological clinical exam in women with negative tests (103). Most of the HPV self-sampling was tested using commercially available HC2 (18, 103), Cobas (15), and other PCR-based methods, particularly RT-PCR (101, 102, 104, 105, 107, 108), and PCR-based testing is preferred to HC2 as it is more sensitive (108). Because the viral load in the vagina is lower than the cervix, a test with high analytic sensitivity appears to be required for self-sampling to ensure equivalent accuracy between clinician and self-sampled specimens (108). It has been demonstrated that ddPCR exhibits high sensitivity, accuracy, specificity, repeatability, and reproducibility compared to RT-PCR in quantifying HPV DNA (31, 71, 92), and therefore it can be used to test the self-sampled swabs. Since ddPCR method is reported to be accurate, repeatable, reproducible (27, 94, 100), and cost-effective (23, 25, 26, 90), it is an ideal method for routine diagnostic testing.

## Conclusion

The routine practice for cervical cancer diagnosis is minimally invasive and utilizes liquid-based cytology, followed by HPV testing using commercially available p16 IHC, DNA/RNA ISH, or DNA/RNA PCR. For OPSCC, the main HPV detection method available is for fresh, frozen, or FFPE tissues using p16 IHC and/or DNA/RNA PCR. For other HPV-related HNSCC, however, HPV testing is not a standard procedure. The sinonasal tract is the second anatomic subsite of the head andneck for HPV-related carcinomas and favorable HPV prognosis is unresolved, therefore moreresearch studies is essential to better understand the role of HR HPVs in sinonasal carcinoma.

Self-sampling HPV testing could be used in the future to replace Pap smears and cervical exams as first-line screening for cervical cancer. However, to ensure similar or better accuracy compared to clinician-collected samples, a test with high analytical sensitivity and specificity is required. For HPV-related HNSCC, swabs will be sufficient for diagnosis, without the need for highly invasive tissue biopsy. p16 IHC is the most widely used method due to its availability in laboratories, but the results can be highly variable, as the criteria for interpretation are not standardized. The commercially available HPV testing methods approved for cervical samples, including HC2, Cervista, Aptima, and Cobas 4800, all have comparable sensitivity and specificity. In comparison to cytology and p16 IHC, they have higher sensitivity but lower specificity.

The new generation of HPV assay, such as ddPCR, is highly sensitive and can be performed on non-invasive samples, such as those obtained using swabs. ddPCR has the potential clinical applicability in early HPV detection for screening, diagnosis, and disease surveillance. It has the ability to amplify a target sequence from minimal RNA samples and provides significantly higher precision and sensitivity for specific DNA/RNA compared to traditional PCR.

## Author Contributions

JW designed the manuscript and wrote the first draft. MK and VB edited the first draft. JW and VB edited and revised the manuscript. All authors contributed to the article and approved the submitted version.

## Conflict of Interest

The authors declare that the research was conducted in the absence of any commercial or financial relationships that could be construed as a potential conflict of interest.

## Publisher’s Note

All claims expressed in this article are solely those of the authors and do not necessarily represent those of their affiliated organizations, or those of the publisher, the editors and the reviewers. Any product that may be evaluated in this article, or claim that may be made by its manufacturer, is not guaranteed or endorsed by the publisher.

## References

[1] Kombe AJK Li B Zahid A Mengist HM Bounda G-A Zhou Y. Epidemiology and Burden of Human Papillomavirus and Related Diseases, Molecular Pathogenesis, and Vaccine Evaluation. Front Public Health (2021) 8:552028. doi: 10.3389/fpubh.2020.552028 33553082 PMC7855977

[2] Plummer M de Martel C Vignat J Ferlay J Bray F Franceschi S. Global Burden of Cancers Attributable to Infections in 2012: A Synthetic Analysis. Lancet Global Health (2016) 4:e609–16. doi: 10.1016/s2214-109x(16)30143-7 27470177

[3] Serrano B Brotons M Bosch FX Bruni L. Epidemiology and Burden of HPV-Related Disease. Best Pract Res Clin Obstet Gynaecol (2018) 47:14–26. doi: 10.1016/j.bpobgyn.2017.08.006 29037457

[4] de Martel C Plummer M Vignat J Franceschi S. Worldwide Burden of Cancer Attributable to HPV by Site, Country and HPV Type. Int J Cancer (2017) 141:664–70. doi: 10.1002/ijc.30716 PMC5520228 28369882

[5] Kilic S Shukla PA Marchiano EJ Patel RH Baredes S Liu JK. Malignant Primary Neoplasms of the Nasal Cavity and Paranasal Sinus. Curr Otorhinolaryngol Rep (2016) 4:249–58. doi: 10.1007/s40136-016-0134-0

[6] Chaturvedi AK D’Souza G Gillison ML Katki HA. Burden of HPV-Positive Oropharynx Cancers Among Ever and Never Smokers in the U.S. Population. Oral Oncol (2016) 60:61–7. doi: 10.1016/j.oraloncology.2016.06.006 PMC11807414 27531874

[7] Dayyani F Etzel CJ Liu M Ho C-H Lippman SM Tsao AS. Meta-Analysis of the Impact of Human Papillomavirus (HPV) on Cancer Risk and Overall Survival in Head and Neck Squamous Cell Carcinomas (HNSCC). Head Neck Oncol (2010) 2:15. doi: 10.1186/1758-3284-2-15 20587061 PMC2908081

[8] Friedman JM Stavas MJ Cmelak AJ. Clinical and Scientific Impact of Human Papillomavirus on Head and Neck Cancer. World J Clin Oncol (2014) 5:781. doi: 10.5306/wjco.v5.i4.781 25302178 PMC4129541

[9] Rahimi S. HPV-Related Squamous Cell Carcinoma of Oropharynx: A Review. J Clin Pathol (2020) 73:624–9. doi: 10.1136/jclinpath-2020-206686 32499224

[10] Antony VM Kakkar A Sikka K Thakar A Deo SVS Bishop JA Jain D. P16 Immunoexpression in Sinonasal and Nasopharyngeal Adenoid Cystic Carcinomas: A Potential Pitfall in Ruling Out HPV‐related Multiphenotypic Sinonasal Carcinoma. Histopathology (2020) 77:989–93. doi: 10.1111/his.14212 32671903

[11] Chen C-C Yang S-F. Human Papillomavirus–Related Carcinoma With Adenoid Cystic–like Features of the Sinonasal Tract (Also Known as Human Papillomavirus–Related Multiphenotypic Sinonasal Carcinoma). Arch Pathol Lab Med (2019) 143:1420–4. doi: 10.5858/arpa.2018-0027-rs 30838880

[12] Burd EM. Human Papillomavirus Detection and Utility of Testing. Clin Microbiol Newsl (2007) 29:159–67. doi: 10.1016/j.clinmicnews.2007.10.001

[13] Burd EM. Human Papillomavirus Laboratory Testing: The Changing Paradigm. Clin Microbiol Rev (2016) 29:291–319. doi: 10.1128/cmr.00013-15 26912568 PMC4786885

[14] Nodjikouambaye ZA Adawaye C Bouassa RM Sadjoli D Bélec L. A Systematic Review of Self‐Sampling for HPV Testing in Africa. Int J Gynecol Obstet (2020) 149:123–9. doi: 10.1002/ijgo.13112 32037532

[15] Ketelaars PJW Bosgraaf RP Siebers AG Massuger LFAG van der Linden JC Wauters CAP. High-Risk Human Papillomavirus Detection in Self-Sampling Compared to Physician-Taken Smear in a Responder Population of the Dutch Cervical Screening: Results of the VERA Study. Prev Med (2017) 101:96–101. doi: 10.1016/j.ypmed.2017.05.021 28579497

[16] Carrasquillo O Seay J Amofah A Pierre L Alonzo Y McCann S. HPV Self-Sampling for Cervical Cancer Screening Among Ethnic Minority Women in South Florida: A Randomized Trial. J Gen Intern Med (2018) 33:1077–83. doi: 10.1007/s11606-018-4404-z PMC6025679 29594933

[17] Sung H Ferlay J Siegel RL Laversanne M Soerjomataram I Jemal A. Global Cancer Statistics 2020: GLOBOCAN Estimates of Incidence and Mortality Worldwide for 36 Cancers in 185 Countries. CA Cancer J Clin (2021) 71:209–49. doi: 10.3322/caac.21660 33538338

[18] Wright JTC Denny L Kuhn L Pollack A Lorincz A. HPV DNA Testing of Self-Collected Vaginal Samples Compared With Cytologic Screening to Detect Cervical Cancer. Jama (2000) 283:81–6. doi: 10.1001/jama.283.1.81 10632284

[19] Walline HM Komarck C McHugh JB Byrd SA Spector ME Hauff SJ. High-Risk Human Papillomavirus Detection in Oropharyngeal, Nasopharyngeal, and Oral Cavity Cancers: Comparison of Multiple Methods. JAMA Otolaryngol Head Neck Surg (2013) 139:1320–7. doi: 10.1001/jamaoto.2013.5460 PMC4049419 24177760

[20] Robinson M Schache A Sloan P Thavaraj S. HPV Specific Testing: A Requirement for Oropharyngeal Squamous Cell Carcinoma Patients. Head Neck Pathol (2012) 6:83–90. doi: 10.1007/s12105-012-0370-7 PMC3394162 22782227

[21] Paver EC Currie AM Gupta R Dahlstrom JE. Human Papilloma Virus Related Squamous Cell Carcinomas of the Head and Neck: Diagnosis, Clinical Implications and Detection of HPV. Pathology (2020) 52:179–91. doi: 10.1016/j.pathol.2019.10.008 31889547

[22] Lewis JSJ Beadle B Bishop JA Chernock RD Colasacco C Lacchetti C. Human Papillomavirus Testing in Head and Neck Carcinomas: Guideline From the College of American Pathologists. Arch Pathol Lab Med (2017) 142:559–97. doi: 10.5858/arpa.2017-0286-cp 29251996

[23] Hindson CM Chevillet JR Briggs HA Gallichotte EN Ruf IK Hindson BJ. Absolute Quantification by Droplet Digital PCR Versus Analog Real-Time PCR. Nat Methods (2013) 10:1003–5. doi: 10.1038/nmeth.2633 PMC4118677 23995387

[24] Hindson BJ Ness KD Masquelier DA Belgrader P Heredia NJ Makarewicz AJ. High-Throughput Droplet Digital PCR System for Absolute Quantitation of DNA Copy Number. Anal Chem (2011) 83:8604–10. doi: 10.1021/ac202028g PMC3216358 22035192

[25] Isaac A Kostiuk M Zhang H Lindsay C Makki F O’Connell DA. Ultrasensitive Detection of Oncogenic Human Papillomavirus in Oropharyngeal Tissue Swabs. J Otolaryngol Head Neck Surg (2017) 46:5. doi: 10.1186/s40463-016-0177-8 28088212 PMC5237494

[26] Biron VL Kostiuk M Isaac A Puttagunta L O’Connell DA Harris J. Detection of Human Papillomavirus Type 16 in Oropharyngeal Squamous Cell Carcinoma Using Droplet Digital Polymerase Chain Reaction. Cancer (2016) 122:1544–51. doi: 10.1002/cncr.29976 26989832

[27] Rotondo JC Oton-Gonzalez L Mazziotta C Lanzillotti C Iaquinta MR Tognon M. Simultaneous Detection and Viral DNA Load Quantification of Different Human Papillomavirus Types in Clinical Specimens by the High Analytical Droplet Digital PCR Method. Front Microbiol (2020) 11:591452. doi: 10.3389/fmicb.2020.591452 33329471 PMC7710522

[28] Hemmat N Bannazadeh Baghi H. Association of Human Papillomavirus Infection and Inflammation in Cervical Cancer. Pathog Dis (2019) 77(5):ftz048. doi: 10.1093/femspd/ftz048 31504464

[29] Tjalmar WAA Waes TRV Eeden LEMV den Bogers JJPM. Role of Human Papillomavirus in the Carcinogenesis of Squamous Cell Carcinoma and Adenocarcinoma of the Cervix. Best Pract Res Cl Ob (2005) 19:469–83. doi: 10.1016/j.bpobgyn.2005.02.002 16150388

[30] Berman TA Schiller JT. Human Papillomavirus in Cervical Cancer and Oropharyngeal Cancer: One Cause, Two Diseases. Cancer (2017) 123:2219–29. doi: 10.1002/cncr.30588 28346680

[31] Veyer D Wack M Mandavit M Garrigou S Hans S Bonfils P. HPV Circulating Tumoral DNA Quantification by Droplet‐Based Digital PCR: A Promising Predictive and Prognostic Biomarker for HPV‐associated Oropharyngeal Cancers. Int J Cancer (2020) 147:1222–7. doi: 10.1002/ijc.32804 31756275

[32] Biron VL Côté DWJ Seikaly H. Oropharyngeal Squamous Cell Carcinoma and Human Papillomavirus-Associated Cancers in Women: Epidemiologic Evaluation of Association. J Otolaryngol Head Neck Surg (2011) 40 Suppl 1:S65–9. doi: 10.2310/7070.2010.100093 21453664

[33] Rahman QB Iocca O Kufta K Shanti RM. Global Burden of Head and Neck Cancer. Oral Maxillofac Surg Clin North Am (2020) 32:367–75. doi: 10.1016/j.coms.2020.04.002 32482563

[34] Bishop JA Guo TW Smith DF Wang H Ogawa T Pai SI. Human Papillomavirus-related Carcinomas of the Sinonasal Tract. Am J Surg Pathology (2013) 37:185–92. doi: 10.1097/pas.0b013e3182698673 PMC3545097 23095507

[35] Lewis JS Westra WH Thompson LDR Barnes L Cardesa A Hunt JL. The Sinonasal Tract: Another Potential “Hot Spot” for Carcinomas with Transcriptionally-Active Human Papillomavirus. Head Neck Pathology (2014) 8:241–49. doi: 10.1007/s12105-013-0514-4 PMC4126925 24338611

[36] Oliver JR Lieberman SM Tam MM Liu CZ Li Z Hu KS. Human Papillomavirus and Survival of Patients with Sinonasal Squamous Cell Carcinoma. Cancer (2020) 126:1413–23. doi: 10.1002/cncr.32679 31886908

[37] Pan C Issaeva N Yarbrough WG. HPV-Driven Oropharyngeal Cancer: Current Knowledge of Molecular Biology and Mechanisms of Carcinogenesis. Cancers Head Neck (2018) 3:12. doi: 10.1186/s41199-018-0039-3 31093365 PMC6460765

[38] Oyervides-Muñoz MA Pérez-Maya AA Rodríguez-Gutiérrez HF Macias GSG Fajardo-Ramírez OR Treviño V. Understanding the HPV Integration and its Progression to Cervical Cancer. Infect Genet Evol (2018) 61:134–44. doi: 10.1016/j.meegid.2018.03.003 29518579

[39] Araldi RP Sant’Ana TA Módolo DG de Melo TC Spadacci-Morena DD Stocco R de C. The Human Papillomavirus (HPV)-Related Cancer Biology: An Overview. BioMed Pharmacother (2018) 106:1537–56. doi: 10.1016/j.biopha.2018.06.149 30119229

[40] Gupta S Kumar P Das BC. HPV: Molecular Pathways and Targets. Curr Probl Cancer (2018) 42:161–74. doi: 10.1016/j.currproblcancer.2018.03.003 29706467

[41] Sano D Oridate N. The Molecular Mechanism of Human Papillomavirus-Induced Carcinogenesis in Head and Neck Squamous Cell Carcinoma. Int J Clin Oncol (2016) 21:819–26. doi: 10.1007/s10147-016-1005-x 27339270

[42] Leemans CR Snijders PJF Brakenhoff RH. The Molecular Landscape of Head and Neck Cancer. Nat Rev Cancer (2018) 18:269–82. doi: 10.1038/nrc.2018.11 29497144

[43] Scarth JA Patterson MR Morgan EL Macdonald A. The Human Papillomavirus Oncoproteins: A Review of the Host Pathways Targeted on the Road to Transformation. J Gen Virol (2021) 102:1540. doi: 10.1099/jgv.0.001540 PMC8148304 33427604

[44] LeConte BA Szaniszlo P Fennewald SM Lou DI Qiu S Chen N-W. Differences in the Viral Genome Between HPV-Positive Cervical and Oropharyngeal Cancer. PloS One (2018) 13:e0203403. doi: 10.1371/journal.pone.0203403 30161236 PMC6117069

[45] Cosper PF Bradley S Luo L Kimple RJ. Biology of HPV Mediated Carcinogenesis and Tumor Progression. Semin Radiat Oncol (2021) 31:265–73. doi: 10.1016/j.semradonc.2021.02.006 PMC8409095 34455982

[46] Re M Gioacchini FM Bajraktari A Tomasetti M Kaleci S Rubini C. Malignant Transformation of Sinonasal Inverted Papilloma and Related Genetic Alterations: A Systematic Review. Eur Arch Otorhinolaryngol (2017) 274:2991–3000. doi: 10.1007/s00405-017-4571-2 28432463

[47] Alvarado-Cabrero I Parra-Herran C Stolnicu S Roma A Oliva E Malpica A. The Silva Pattern-based Classification for HPV-associated Invasive Endocervical Adenocarcinoma and the Distinction Between In Situ and Invasive Adenocarcinoma: Relevant Issues and Recommendations From the International Society of Gynecological Pathologists. Int J Gynecol Pathol (2021) 40:S48–S65. Doi: 10.1097/pgp.0000000000000735 33570863 PMC7969170

[48] Höhn AK Brambs CE Hiller GGR May D Schmoeckel E Horn L-C. WHO Classification of Female Genital Tumors. Geburtsh Frauenheilk (2021) 81:1145–53. Doi: 10.1055/a-1545-4279 PMC8494521 34629493

[49] Dogan S Xu B Middha S Vanderbilt CM Bowman AS Migliacci J. Identification of Prognostic Molecular Biomarkers in 157 HPV‐positive and HPV‐negative Squamous Cell Carcinomas of the Oropharynx. Int J Cancer (2019) 145:3152–62. doi: 10.1002/ijc.32412 PMC7595146 31093971

[50] Sabatini ME Chiocca S. Human Papillomavirus as a Driver of Head and Neck Cancers. Br J Cancer (2020) 122:306–14. doi: 10.1038/s41416-019-0602-7 PMC7000688 31708575

[51] Devins KM Tetzlaff MT Baloch Z LiVolsi VA. The Evolving Landscape of HPV-Related Neoplasia in the Head and Neck. Hum Pathol (2019) 94:29–39. doi: 10.1016/j.humpath.2019.09.001 31654690

[52] Lewis JS. Sinonasal Squamous Cell Carcinoma: A Review with Emphasis on Emerging Histologic Subtypes and the Role of Human Papillomavirus. Head Neck Pathol (2016) 10:60–67. Doi: 10.1007/s12105-016-0692-y 26830402 PMC4746138

[53] Bishop JA Andreasen S Hang J-F Bullock MJ Chen TY Franchi A. HPV-Related Multiphenotypic Sinonasal Carcinoma. Am J Surg Pathol (2017) 41:1690–701. doi: 10.1097/pas.0000000000000944 PMC5680105 28877065

[54] Ward ML Kernig M Willson TJ. HPV‐Related Multiphenotypic Sinonasal Carcinoma: A Case Report and Literature Review. Laryngoscope (2021) 131:106–10. doi: 10.1002/lary.28598 32159863

[55] Bishop JA Ogawa T Stelow EB Moskaluk CA Koch WM Pai SI. Human Papillomavirus–related Carcinoma With Adenoid Cystic–like Features. Am J Surg Pathol (2013) 37:836–44. doi: 10.1097/pas.0b013e31827b1cd6 PMC3653997 23598962

[56] Thompson LDR Franchi A. New Tumor Entities in the 4th Edition of the World Health Organization Classification of Head and Neck Tumors: Nasal Cavity, Paranasal Sinuses and Skull Base. Virchows Arch (2018) 472:315–30. doi: 10.1007/s00428-017-2116-0 28444451

[57] Pang KJWCS Mur T Collins L Rao SR Faden DL. Human Papillomavirus in Sinonasal Squamous Cell Carcinoma: A Systematic Review and Meta-Analysis. Cancers (2020) 13:45. doi: 10.3390/cancers13010045 33561073 PMC7796014

[58] Famuyide A Juliano A Moonis G. MRI of Sinonasal Malignancies. Top Magn Reson Imag (2021) 30:139–49. doi: 10.1097/rmr.0000000000000288 34096897

[59] HPV Vaccine: Canadian Immunization Guide. Available at: https://www.canada.ca/en/public-health/services/publications/healthy-living/canadian-immunization-guide-part-4-active-vaccines/page-9-human-papillomavirus-vaccine.html#a5.

[60] El-Zein M Richardson L Franco EL. Cervical Cancer Screening of HPV Vaccinated Populations: Cytology, Molecular Testing, Both or None. J Clin Virol (2016) 76:S62–8. doi: 10.1016/j.jcv.2015.11.020 PMC4789074 26631958

[61] Sawaya GF Smith-McCune K Kuppermann M. Cervical Cancer Screening. Jama (2019) 321:2018–9. doi: 10.1001/jama.2019.4595 PMC6656358 31135834

[62] Höffken H Soost H. Cervical Cytology as a Screening Method. Curr Top Pathol (1981) 70:21–65. doi: 10.1007/978-3-642-68185-1 7297133

[63] Doorbar J Egawa N Griffin H Kranjec C Murakami I. Human Papillomavirus Molecular Biology and Disease Association. Rev Med Virol (2015) 25:2–23. doi: 10.1002/rmv.1822 25752814 PMC5024016

[64] Wang X Huang X Zhang Y. Involvement of Human Papillomaviruses in Cervical Cancer. Front Microbiol (2018) 9:2896. doi: 10.3389/fmicb.2018.02896 30546351 PMC6279876

[65] Bhatla N Singhal S. Primary HPV Screening for Cervical Cancer. Best Pract Res Clin Obstet Gynaecol (2020) 65:98–108. doi: 10.1016/j.bpobgyn.2020.02.008 32291178

[66] Wright TC Stoler MH Behrens CM Sharma A Zhang G Wright TL. Primary Cervical Cancer Screening With Human Papillomavirus: End of Study Results From the ATHENA Study Using HPV as the First-Line Screening Test. Gynecol Oncol (2015) 136:189–97. doi: 10.1016/j.ygyno.2014.11.076 25579108

[67] Zhang S Batur P. Human Papillomavirus in 2019: An Update on Cervical Cancer Prevention and Screening Guidelines. Cleve Clin J Med (2019) 86:173–8. doi: 10.3949/ccjm.86a.18018 30849035

[68] Prigge E Arbyn M von Knebel Doeberitz M Reuschenbach M. Diagnostic Accuracy of P16ink4a Immunohistochemistry in Oropharyngeal Squamous Cell Carcinomas: A Systematic Review and Meta‐Analysis. Int J Cancer (2017) 140:1186–98. doi: 10.1002/ijc.30516 27859245

[69] Thomas J Primeaux T. Is P16 Immunohistochemistry a More Cost-Effective Method for Identification of Human Papilloma Virus–Associated Head and Neck Squamous Cell Carcinoma? Ann Diagn Pathol (2012) 16:91–9. doi: 10.1016/j.anndiagpath.2011.09.002 22197546

[70] Aziz S Mehta R. Technical Aspects of Toxicological Immunohistochemistry, System Specific Biomarkers. Springer New York (2016). doi: 10.1007/978-1-4939-1516-3

[71] Augustin JG Lepine C Morini A Brunet A Veyer D Brochard C. HPV Detection in Head and Neck Squamous Cell Carcinomas: What Is the Issue? Front Oncol (2020) 10:1751. doi: 10.3389/fonc.2020.01751 33042820 PMC7523032

[72] Larsen CG Gyldenløve M Kiss K Buchwald C. Who Evaluates P16 Immunohistochemistry? Apmis (2015) 123:912–3. doi: 10.1111/apm.12421 26177668

[73] Jensen E. Technical Review: In Situ Hybridization. Anat Rec (2014) 297 (8):1349–53. doi: 10.1002/ar.22944 24810158

[74] Venuti A Paolini F. HPV Detection Methods in Head and Neck Cancer. Head Neck Pathol (2012) 6:63–74. doi: 10.1007/s12105-012-0372-5 PMC3394157 22782225

[75] Waters D Shapter FM. The Polymerase Chain Reaction (PCR): General Methods. Methods Mol Biol (2014) 1099:65–75. doi: 10.1007/978-1-62703-715-0 24243196

[76] Shampo MA Kyle RA Mullis KB. Nobel Laureate for Procedure to Replicate DNA. Mayo Clin Proc (2002) 77:606. doi: 10.4065/77.7.606 12108595

[77] Pagliusi SR Garland SM. International Standard Reagents for HPV Detection. Dis Markers (2007) 23:283–96. doi: 10.1155/2007/591826 PMC3851336 17627063

[78] Westra WH. Detection of Human Papillomavirus in Clinical Samples. Otolaryngol Clin North Am (2012) 45(4):765–77. doi: 10.1016/j.otc.2012.04.001 22793851

[79] Laudadio J. Human Papillomavirus Detection: Testing Methodologies and Their Clinical Utility in Cervical Cancer Screening. Adv Anatomic Pathol (2013) 20 (3):158–67. doi: 10.1097/pap.0b013e31828d1893 23574772

[80] Westra WH. Detection of Human Papillomavirus (HPV) in Clinical Samples: Evolving Methods and Strategies for the Accurate Determination of HPV Status of Head and Neck Carcinomas. Oral Oncol (2014) 50:771–9. doi: 10.1016/j.oraloncology.2014.05.004 PMC4318232 24932529

[81] HOLOGIC. HOLOGIC Aptima HPV Assay. (2017). Available at: https://www.hologic.com/sites/default/files/package-insert/AW-14517-001_003_01.pdf.

[82] ArborVita. OncoE6TM Cervical Test Instructions for Use. (2020). Available at: http://www.arborvita.com/wp-content/uploads/2020/06/LBL2000050_OncoE6-Cervical-Test-Instructions-for-Use_RevN.pdf.

[83] Krings A Dückelmann AM Moser L Gollrad J Wiegerinck M Schweizer J . Performance of OncoE6 Cervical Test With Collection Methods Enabling Self-Sampling. Bmc Women’s Heal (2018) 18:68. doi: 10.1186/s12905-018-0559-3 PMC5963066 29783960

[84] Valdez M Jeronimo J Bansil P Qiao Y Zhao F Chen W. Effectiveness of Novel, Lower Cost Molecular Human Papillomavirus‐Based Tests for Cervical Cancer Screening in Rural China. Int J Cancer (2016) 138:1453–1461. doi: 10.1002/ijc.29877 26421807

[85] Torres KL Mariño JM Rocha DAP de Mello MB de Farah HH dos S Reis R. Self-Sampling Coupled to the Detection of HPV 16 and 18 E6 Protein: A Promising Option for Detection of Cervical Malignancies in Remote Areas. Plos One (2018) 13:e0201262. doi: 10.1371/journal.pone.0201262 30036381 PMC6056043

[86] Jiromaru R Yamamoto H Yasumatsu R Hongo T Nozaki Y Hashimoto K. HPV-Related Sinonasal Carcinoma. Am J Surg Pathol (2020) 44:305–15. doi: 10.1097/pas.0000000000001410 31743130

[87] Švajdler M Němcova J Dubinský P Metelkova A Švajdler P Straka Ľ. Significance of Transcriptionally-Active High-Risk Human Papillomavirus in Sinonasal Squamous Cell Carcinoma: Case Series and a Meta-Analysis. Neoplasma (2021) 67:1456–63. doi: 10.4149/neo_2020_200330n332 32853018

[88] Menegaldo A Schroeder L Holzinger D Tirelli G Cin ED Tofanelli M. Detection of HPV16/18 E6 Oncoproteins in Head and Neck Squamous Cell Carcinoma Using a Protein Immunochromatographic Assay. Laryngoscope (2021) 131:1042–8. doi: 10.1002/lary.29184 33103777

[89] Chernesky M Jang D Schweizer J Arias M Doerwald-Munoz L Gupta M. HPV E6 Oncoproteins and Nucleic Acids in Neck Lymph Node Fine Needle Aspirates and Oral Samples From Patients With Oropharyngeal Squamous Cell Carcinoma. Papillomavirus Res (2018) 6:1–5. doi: 10.1016/j.pvr.2018.05.003 29842928 PMC5986165

[90] Damerla RR Lee NY You D Soni R Shah R Reyngold M. Detection of Early Human Papillomavirus–Associated Cancers by Liquid Biopsy. JCO Precis Oncol (2019) 3:18:00276. doi: 10.1200/po.18.00276 PMC6726127 31485558

[91] Jeannot E Latouche A Bonneau C Calméjane M-A Beaufort CM Ruigrok-Ritstier K. Circulating HPV DNA as a Marker for Early Detection of Relapse in Patients With Cervical Cancer. Clin Cancer Res (2021) 27:clincanres.0625.2021. doi: 10.1158/1078-0432.ccr-21-0625 PMC9401545 34210686

[92] Jeannot E Becette V Campitelli M Calméjane M Lappartient E Ruff E. Circulating Human Papillomavirus DNA Detected Using Droplet Digital PCR in the Serum of Patients Diagnosed With Early Stage Human Papillomavirus‐Associated Invasive Carcinoma. J Pathol Clin Res (2016) 2:201–9. doi: 10.1002/cjp2.47 PMC5129558 27917295

[93] BIORAD. Droplet DigitalTM PCR Applications Guide (2011). Available at: https://www.bio-rad.com/webroot/web/pdf/lsr/literature/Bulletin_6407.pdf.

[94] Taylor SC Laperriere G Germain H. Droplet Digital PCR Versus qPCR for Gene Expression Analysis With Low Abundant Targets: From Variable Nonsense to Publication Quality Data. Sci Rep-uk (2017) 7:2409. doi: 10.1038/s41598-017-02217-x PMC5445070 28546538

[95] Li H Bai R Zhao Z Tao L Ma M Ji Z. Application of Droplet Digital PCR to Detect the Pathogens of Infectious Diseases. Biosci Rep (2018) 38:BSR20181170. doi: 10.1042/bsr20181170 30341241 PMC6240714

[96] Malin K Louise BM Gisela H Mats KG Gabriella L-L. Optimization of Droplet Digital PCR Assays for the Type-Specific Detection and Quantification of Five HPV Genotypes, Including Additional Data on Viral Loads of Nine Different HPV Genotypes in Cervical Carcinomas. J Virol Methods (2021) 294:114193. doi: 10.1016/j.jviromet.2021.114193 34022300

[97] Larsson GL Helenius G. Digital Droplet PCR (ddPCR) for the Detection and Quantification of HPV 16, 18, 33 and 45 - a Short Report. Cell Oncol (Dordr) (2017) 40:521–7. doi: 10.1007/s13402-017-0331-y PMC5608796 28748500

[98] Schiavetto CM de Abreu PM von Zeidler SV de Jesus LM Carvalho RS Cirino MT. Human Papillomavirus DNA Detection by Droplet Digital PCR in Formalin-Fixed Paraffin-Embedded Tumor Tissue From Oropharyngeal Squamous Cell Carcinoma Patients. Mol Diagn Ther (2021) 25:59–70. doi: 10.1007/s40291-020-00502-6 33245553

[99] Antonsson A Knight L Panizza BJ Porceddu SV Emmett S Whiteman DC. HPV-16 Viral Load in Oropharyngeal Squamous Cell Carcinoma Using Digital PCR. Acta Otolaryngol (2018) 138:1–5. doi: 10.1080/00016489.2018.1461239 29741428

[100] Rocca FL Grieco V Ruggieri V Zifarone E Villani O Zoppoli P. Superiority of Droplet Digital PCR Over Real-Time Quantitative PCR for JAK2 V617F Allele Mutational Burden Assessment in Myeloproliferative Neoplasms: A Retrospective Study. Diagnostics (2020) 10:143. doi: 10.3390/diagnostics10030143 PMC7151190 32150880

[101] Schmeink CE Bekkers RLM. Implementation of HPV Self-Sampling in Cervical Screening Programs Increases Participation Rates: A Literature Review. Indian J Gynecol Oncol (2021) 19:54. doi: 10.1007/s40944-021-00539-3

[102] Bosgraaf RP Verhoef VMJ Massuger LFAG Siebers AG Bulten J de Kuyper-de Ridder GM. Comparative Performance of Novel Self‐Sampling Methods in Detecting High‐Risk Human Papillomavirus in 30,130 Women Not Attending Cervical Screening. Int J Cancer (2015) 136:646–55. doi: 10.1002/ijc.29026 24923998

[103] Sancho‐Garnier H Tamalet C Halfon P Leandri FX Retraite LL Djoufelkit K. HPV Self‐Sampling or the Pap‐smear: A Randomized Study Among Cervical Screening Nonattenders From Lower Socioeconomic Groups in France. Int J Cancer (2013) 133:2681–7. doi: 10.1002/ijc.28283 23712523

[104] Gustavsson I Aarnio R Berggrund M Hedlund-Lindberg J Strand A-S Sanner K. Randomised Study Shows That Repeated Self-Sampling and HPV Test has More Than Two-Fold Higher Detection Rate of Women With CIN2+ Histology Than Pap Smear Cytology. Br J Cancer (2018) 118:896–904. doi: 10.1038/bjc.2017.485 29438367 PMC5886121

[105] Zehbe I Jackson R Wood B Weaver B Escott N Severini A. Community-Randomised Controlled Trial Embedded in the Anishinaabek Cervical Cancer Screening Study: Human Papillomavirus Self-Sampling Versus Papanicolaou Cytology. BMJ Open (2016) 6:e011754. doi: 10.1136/bmjopen-2016-011754 PMC5073481 27855089

[106] Oketch SY Kwena Z Choi Y Adewumi K Moghadassi M Bukusi EA. Perspectives of Women Participating in a Cervical Cancer Screening Campaign With Community-Based HPV Self-Sampling in Rural Western Kenya: A Qualitative Study. BMC Womens Health (2019) 19:75. doi: 10.1186/s12905-019-0778-2 31196175 PMC6567898

[107] Moses E Pedersen HN Mitchell SM Sekikubo M Mwesigwa D Singer J. Uptake of Community‐Based, Self‐Collected HPV Testing vs. Visual Inspection With Acetic Acid for Cervical Cancer Screening in Kampala, Uganda: Preliminary Results of a Randomised Controlled Trial. Trop Med Int Health (2015) 20:1355–67. doi: 10.1111/tmi.12549 26031572

[108] Haguenoer K Sengchanh S Gaudy-Graffin C Boyard J Fontenay R Marret H. Vaginal Self-Sampling is a Cost-Effective Way to Increase Participation in a Cervical Cancer Screening Programme: A Randomised Trial. Br J Cancer (2014) 111:2187–96. doi: 10.1038/bjc.2014.510 PMC4260034 25247320

